# Structurally Related Liposomes Containing *N*-Oxide Surfactants: Physicochemical Properties and Evaluation of Antimicrobial Activity in Combination with Therapeutically Available Antibiotics

**DOI:** 10.1021/acs.molpharmaceut.1c00609

**Published:** 2022-02-16

**Authors:** Sara Battista, Pierangelo Bellio, Lorenza Fagnani, Elena Allegritti, Lisaurora Nazzicone, Luciano Galantini, Giuseppe Celenza, Luisa Giansanti

**Affiliations:** † Dipartimento di Scienze Fisiche e Chimiche, Università degli Studi dell’Aquila, Via Vetoio 10, 67010 Coppito, AQ, Italy; ‡ Dipartimento di Scienze Chimiche Applicate e Biotecnologie, Università degli Studi dell’Aquila, Via Vetoio 10, 67010 Coppito, AQ, Italy; § Dipartimento di Chimica, Università degli Studi di Roma “Sapienza”, P.le A. Moro 5, 00185 Roma, Italy

**Keywords:** liposomes, structure−activity relation, antibacterial activity, l-prolinol derivatives, synergistic effect

## Abstract

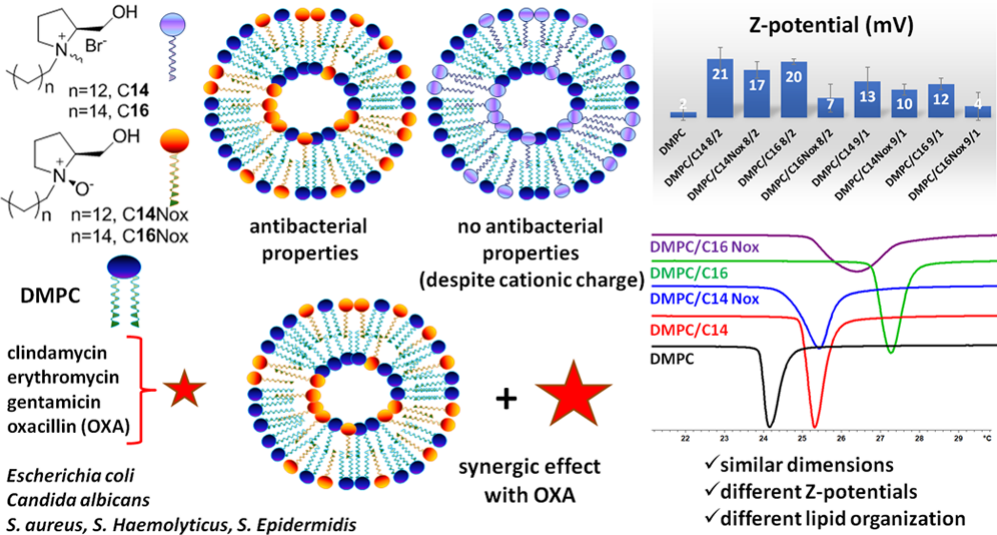

Although liposomes are largely investigated as drug delivery systems, they can also exert a pharmacological activity if devoid of an active principle as a function of their composition. Specifically, charged liposomes can electrostatically interact with bacterial cells and, in some cases, induce bacterial cell death. Moreover, they also show a high affinity toward bacterial biofilms. We investigated the physicochemical and antimicrobial properties of liposomes formulated with a natural phospholipid and four synthetic l-prolinol-derived surfactants at 9/1 and 8/2 molar ratios. The synthetic components differ in the nature of the polar headgroup (quaternary ammonium salt or *N*-oxide) and/or the length of the alkyl chain (14 or 16 methylenes). These differences allowed us to investigate the effect of the molecular structure of liposome components on the properties of the aggregates and their ability to interact with bacterial cells. The antimicrobial properties of the different formulations were assessed against Gram-negative and Gram-positive bacteria and fungi. Drug–drug interactions with four classes of available clinical antibiotics were evaluated against *Staphylococcus* spp. The target of each class of antibiotics plays a pivotal role in exerting a synergistic effect. Our results highlight that the liposomal formulations with an *N*-oxide moiety are required for the antibacterial activity against Gram-positive bacteria. In particular, we observed a synergism between oxacillin and liposomes containing 20 molar percentage of *N*-oxide surfactants on*Staphylococcus haemolyticus*, *Staphylococcus epidermidis*, and*Staphylococcus aureus*. In the case of liposomes containing 20 molar percentage of the *N*-oxide surfactant with 14 carbon atoms in the alkyl chain for *S. epidermidis*, the minimum inhibitory concentration was 0.125 μg/mL, well below the breakpoint value of the antibiotic.

## 1 Introduction

Liposomes are vesicular aggregates largely investigated for their potentiality in many fields. They are optimal candidates as drug delivery systems because they can entrap both hydrophobic and hydrophilic molecules (influencing their pharmacokinetics and pharmacodynamics). In addition, they are biocompatible and the possibility to functionalize their surface facilitates the interaction with the target tissue. The first liposomal formulation reached the market in the ’90s and included the anticancer drug doxorubicin (Doxil)^1^ or the antifungal drug amphotericin B (Ambisome).^2^ Many liposome-based drug formulations are currently available and many others are under clinical trials, thanks to the extensive progress in liposome technology.^3^

Liposomal properties are strictly related to their composition. It is well known that cationic liposomes containing quaternary ammonium surfactants show antibacterial,^4,5^ antifungal,^6,7^ and antiviral^8^ activity. The latter is strictly related to the molecular structure of the surfactant incorporated in the formulation.^9,10^ For instance, a pyrrolidinium ring imparts peculiar properties to the molecules and their aggregates, influencing the hydration, volume and topology of the polar headgroup.^11−13^ Literature reports confirm that natural or synthetic lipids can feature antimicrobial activity: the presence and position of an unsaturation and the chain length are crucial factors in determining the antimicrobial activity of lipids.^14−17^ Among all of the categories of surfactants, the *N*-oxide ones feature very attractive properties because of their low or absent toxicity,^18^ biodegradability,^19^ pH-sensitive aggregative behavior and performances in different fields.^20^ Moreover, they are very easy to prepare and are environmentally friendly.^21^ Based on these premises, *N*-oxide surfactants are used in many everyday products such as hair and body care products and dish and laundry detergents.^22^ These surfactants can form different supramolecular aggregates and it is possible to tune their properties by selective modification of their chemical lipid composition. Moreover, surfactants bearing the *N*-oxide moiety can prevent protein–protein interaction^23^ and confer antibacterial or antioxidant properties to the aggregates, depending on their molecular scaffold.^24,25^

Because of their intrinsic antimicrobial activity, some liposome formulations^4−8^ can be exploited as potential adjuvants in antimicrobial therapy, specifically against those microorganisms expressing a multidrug-resistant (MDR) phenotype. MDR pathogens are organisms capable of resisting the action of several classes of antibiotics, making their use ineffective in treating infectious diseases in nosocomial and community settings. Nowadays, the phenomenon of antimicrobial resistance (AMR) has assumed the character of a global emergency and health care systems face one of the most significant crises due to the dramatic increase in mortality, human sufferance and economic loss. Therefore, identifying new strategies to overcome the phenomenon of AMR is mandatory, specifically if we consider that the pipeline in the development of new drugs is to a dead end and the identification of new potential targets is far from being real.

Today, the use of antimicrobial adjuvants seems to be the most feasible strategy to fight antimicrobial resistance. The use of antibiotic potentiators, also known as “antibiotic resistance breakers” (ARB) capable of re-sensitizing resistant bacteria to antibiotics, is a practicable and solid approach to revitalize old and no more efficacious antimicrobials. The most important classes of ARBs are membrane permeabilizers, modifying-enzyme inhibitors (such as the β-lactam inhibitors), and efflux pump inhibitors.^26^ In general, these compounds increase the effectiveness of the antibiotic treatment, undermining the resistance mechanisms responsible for AMR (without stimulating host defense mechanisms).^27^ ARBs exert a direct antibacterial action that supports the eradication of bacterial infections promoted by the antibiotic. The idea of coadministering ARBs with failing antibiotics results from the synergistic and/or additive effects of dual antibiotic therapy.^28^ A successful ARB allows the reduction of the dose of the antibiotic to be administered with respect to antibiotic monotherapy, with a consequent slowdown of the onset of AMR and reduction of side effects.

**Chart 1:**
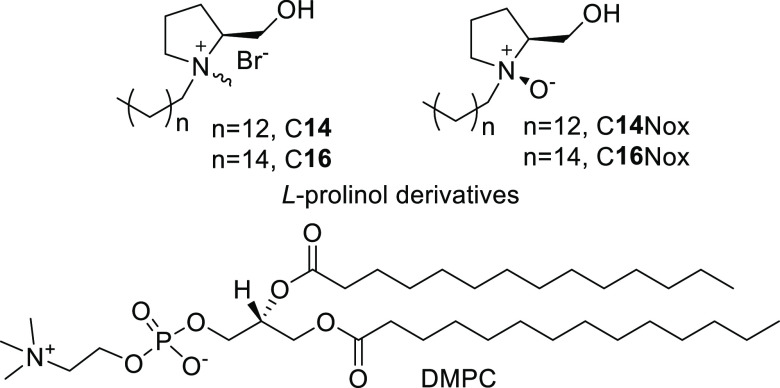
Liposome Components

Here we report an investigation on the physicochemical and antimicrobial properties of liposomes composed of a saturated natural phospholipid, the 1,2-dimyristoyl-*sn*-glycero-3-phosphocholine (DMPC), and one of the l-prolinol-derived surfactants, reported in Chart 1, at different molar ratios. In addition, the effect of different chain lengths (C14 and C16) and the presence of an *N*-oxide or a quaternary ammonium moiety were evaluated. In previous investigations, we reported the biophysical and antibacterial properties of micelles formed by the investigated synthetic *N*-oxide-based surfactants.^24,25,29−31^

Moreover, we also demonstrated that the structural features of these compounds could significantly affect liposome properties and, consequently, their ability to interact with target cells.^32,33^ The antibacterial properties of the different formulations were assessed in combination with several antibiotics against a panel of Gram-negative, Gram-positive bacterial strains and fungi.

## 2 Experimental Section

### 2.1 Materials

DMPC, phosphate-buffered saline tablets (PBS, 0.01 M phosphate buffer; 0.0027 M KCl; 0.137 M NaCl; pH 7.4), and all tested antibiotics (clindamycin (CLI), erythromycin (ERY), gentamicin (GEN), and oxacillin (OXA)) were purchased from Sigma-Aldrich (Milan, Italy). The synthetic surfactants were prepared as previously described.^24^ All reagents and solvents were used without further purification.

### 2.2 Organisms

The standard organisms *Staphylococcus aureus* ATCC 43300 with methicillin-resistant profile, *Escherichia coli* ATCC 25922, and *Candida albicans* ATCC 64124 were from Liofilchem (Teramo, Italy), while the clinical strains *S. aureus* 29A, *Staphylococcus haemolyticus* 12H, and *Staphylococcus epidermidis* 20E were collected at the “San Salvatore” Hospital of L’Aquila, Italy. They have been isolated from hospitalized patients, surgical wounds, vascular and urinary catheters, and blood and respiratory tracts.

## 3 Methods

### 3.1 Liposome Preparation

The liposomal formulations reported in Table 1 were prepared in sterile conditions by evaporation inside the wall of a round-bottom flask of solutions containing a certain amount of DMPC and one of the l-prolinol derivatives, in the chosen molar ratio, both previously dissolved in CHCl_3_. The obtained films were kept overnight under reduced pressure (0.4 mbar) to remove the solvent residues; then PBS was added to obtain a 10 mM lipid dispersion of multilamellar vesicles (MLV). Next, the solutions were heated at 50 °C and vortex-mixed. The suspensions were sonicated for 12 min at 72 W (cycles: 0.5 s) in an ice-water bath, using a Hielscher UP100-H ultrasonic processor with a microtip probe (7 mm).

**Table 1:** Liposomal Formulations

Liposomal Formulation	Ratio
DMPC	10/0
DMPC/C**14**	8/2
DMPC/C**14**Nox	8/2
DMPC/C**16**	8/2
DMPC/C**16**Nox	8/2
DMPC/C**14**	9/1
DMPC/C**14**Nox	9/1
DMPC/C**16**	9/1
DMPC/C**16**Nox	9/1

### 3.2 DLS and *Z*-Potential Measurements

DLS and electrophoretic mobility measurements employing the laser Doppler electrophoresis technique were carried out at 25 °C on 1 mM liposome solutions soon after their preparation, using a Malvern Zetasizer apparatus equipped with a 5 mW He–Ne laser operating at 633 nm and a digital logarithmic correlator. The measured autocorrelation functions were analyzed using the non-negative least square (NNLS) algorithm to obtain the size distribution. The distribution of the diffusion coefficients *D* of the particles was converted into the distribution of the apparent hydrodynamic diameters *D*_H_ using the Stokes–Einstein relationship *D*_H_ = *kT*/3πη*D*, where *kT* is the thermal energy and η is the solvent viscosity. The reported *D*_H_ values correspond to the average values obtained from the intensity-weighted distributions over several measurements. The electrophoretic mobility measurements to determine the *Z*-potential were carried out through the laser Doppler electrophoresis technique. Analysis of the Doppler shift in the Zetasizer Nano series was done using phase analysis light scattering (PALS) implemented with M3 (mixed mode measurement). *Z*-potential was inferred from the electrophoretic mobility under the Smoluchowsky approximation. Low applied voltages were used to avoid the risk of effects due to Joule heating. All of the reported values were the averages of three consecutive measurements of three independent samples. Excel was used to evaluate the significance of differences between group means by one-way analysis of variance (one-way ANOVA) with a 95% confidence interval (*p* < 0.05 was considered to be statistically significant).

### 3.3 Liposome Morphology

A scanning electron microscope (ZEISS GeminiSEM 500) with an annular detector, aSTEM, was used to observe the morphology and dimensions of 1 mM liposomes soon after their preparation. Briefly, 10 μL of the investigated liposome suspensions was air-dried onto a copper grid for electron microscopy, covered by a thin amorphous carbon film.

### 3.4 Determination of the Thermotropic Properties of Liposomes

Differential scanning calorimetry (DSC) measurements were carried out on 30 μL of MLV using a Mettler Toledo DSC 3 calorimeter. Aluminum pans of 40 μL and a reference PBS-filled pan were used. Liposomes (1 mg/10 μL, ≈148 mM in total lipids) were prepared in PBS. Two heating and cooling scans were recorded at the rate of 5 °C/min and two subsequent heating and cooling scans were recorded at the rate of 1 °C/min. Under the experimental conditions, reproducible thermal recordings were obtained. The uncertainty on temperatures was ±0.1 °C and that on Δ*H* was ±0.5 kJ/mol.

### 3.5 *In Vitro* Susceptibility Test

The antimicrobial susceptibility tests for the free antibiotics and liposomes were done separately by following the CLSI recommendation.^34^ Briefly, sterile 96-well microdilution plates containing 100 μL of serially diluted antibiotic or liposome formulation in cation-adjusted Mueller–Hinton were inoculated with 100 μL of 10^6^ CFU/mL bacterial suspension in 0.9% saline solution (NaCl) to reach a final volume of 200 μL. Positive control wells were prepared with the culture medium and bacterial suspension, while the negative control wells were prepared with the culture medium and liposomal formulation. The microdilution plates were incubated for 18 h at 37 °C. When OXA was tested, 2% NaCl was added to the cation-adjusted Mueller–Hinton, and the microplates were incubated for 24 h at 37 °C. The growth in each well was quantified spectrophotometrically at 595 nm by the microplate reader iMark, BioRad (Milan, Italy). The minimum inhibitory concentration (MIC) was defined as the concentration of the drug that reduces the growth by 80%, compared to that of organisms grown in the absence of the drug. The MIC value was determined as the median of at least three independent experiments and the data reported in this study are expressed as mean ± standard error (SE).

### 3.6 Checkerboard Microdilution Assay

As previously described, the *in vitro* tests for evaluating the interactions between the free antibiotics and the liposomal formulations added separately were assessed through the checkerboard microdilution assay.^35,36^ The microplates were incubated at 37 °C for 18 h, while for OXA, the incubation was 24 h. The growth in each well was spectrophotometrically quantified at 595 nm using the microplate reader iMark, BioRad (Milan, Italy). The percentage of growth in each well was calculated as the ratio of the OD_595_ of each well to the OD_595_ of the drug-free well, after subtraction of the background OD_595_ obtained from the microorganism-free plates. All experiments were performed in triplicate, and the data reported in this study are expressed as mean ± SE.

### 3.7 Drug Interaction Models

To assess the nature of the *in vitro* interactions between the liposomal formulations and antibiotics against the various microbial strains, the data obtained from the checkerboard microdilution assay were investigated through two different nonparametric interpretative models, based on the Loewe additivity model (LA) and Bliss’s theory of independence (BI) as previously described.^35,37,38^

## 4 Results

### 4.1 Liposome Dimensions, Morphology, and *Z*-Potential

**Figure 1:**
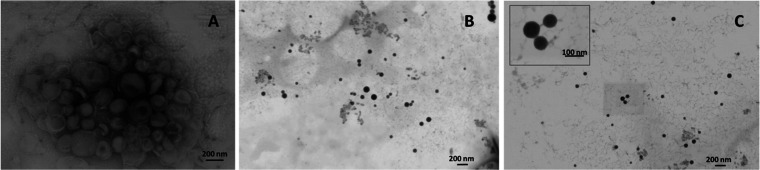
(A) SEM image of DMPC MLV; (B) SEM image of DMPC/C**14**Nox-sonicated liposomes at 9/1 molar ratio; and (C) SEM image of DMPC/C**14**Nox-sonicated liposomes at 8/2 molar ratio.

The size distributions of all of the investigated formulations exhibited the main peak at *D*_H_ values of ∼80 to 90 nm (Table 2) and a minor population (less than 5%) with dimensions in the range 0.5–1 μm. In all cases, the statistical analysis showed that there were no significant differences (*p* 0.13, thus > 0.05) among the average values of dimensions of the three independent samples (Table 2). The liposome morphology was confirmed by SEM analysis. Some representative images of MLV (before sonication) composed of mere DMPC and of mixed sonicated unilamellar liposomes are shown in Figure 1. These images confirm that before sonication, liposomes are heterogeneous for multilamellarity and size (Figure 1A) whereas upon sonication, unilamellar monodisperse liposomes (whose dimensions are in pretty good agreement with the DLS results reported in Table 2) can be clearly observed.

**Table 2:** NNLS Main Peak Diameters (nm) and Average Z-Potential Values (mV) of the Investigated Liposomal Formulations Obtained by the Three Independent Samples^a^

Formulation	Size^b^ (nm)	*Z*-potential (mV)
DMPC	83 ± 9	+3 ± 3
DMPC/C**14** 8/2	78 ± 7	+21 ± 3^c^
DMPC/C**14**Nox 8/2	99 ± 6	+17 ± 3
DMPC/C**16** 8/2	88 ± 8	+19 ± 2^c^
DMPC/C**16**Nox 8/2	95 ± 15	+8 ± 3
DMPC/C**14** 9/1	94 ± 12	+14 ± 3^c^
DMPC/C**14**Nox 9/1	101 ± 14	+11 ± 3
DMPC/C**16** 9/1	104 ± 10	+13 ± 2^c^
DMPC/C**16**Nox 9/1	90 ± 14	+5 ± 4

a Estimated standard deviations (ESD) are reported.

b The observed differences in size are not significantly different from one-way ANOVA.

c These samples show a statistically significant difference from their counterparts without *N*-oxide moiety.

*Z*-potential of the investigated formulations was also assessed. This parameter can be considered as the potential difference between the dispersion medium and the stationary layer of the fluid attached to the dispersed particle. Results of the one-way ANOVA test demonstrated that the observed differences are significantly different (*p* 2 × 10^–5^, thus < 0.05). The value of the *Z*-potential of liposomes containing a cationic surfactant was positive and increased with their molar percentage. On the other hand, the *Z*-potential slightly decreased for the formulations containing surfactants bearing an *N*-oxide moiety, especially in formulations containing C**16**Nox (Table 2).

### 4.2 Thermotropic Properties of the Liposomes

**Figure 2:**
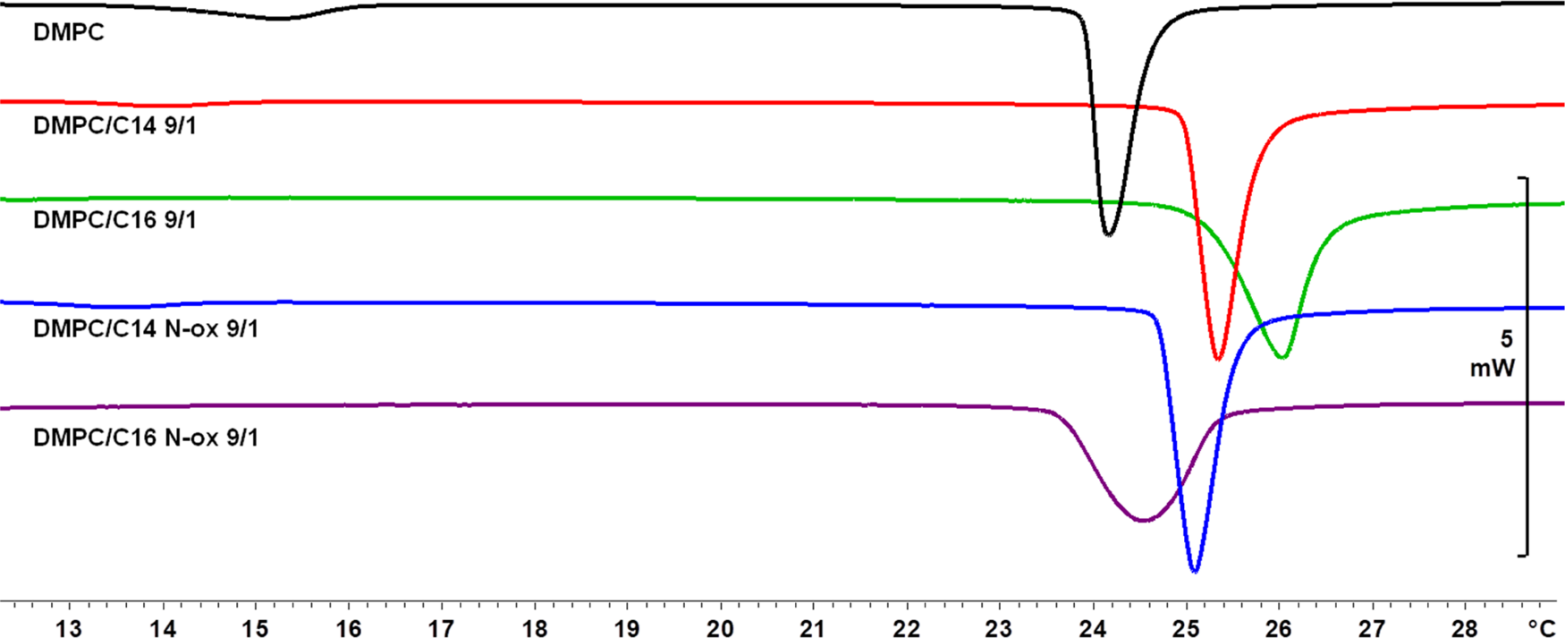
Thermograms of MLV containing DMPC or DMPC/l-prolinol derivative at 9/1 molar ratio. The scan rate is 1 °C/min.

**Figure 3:**
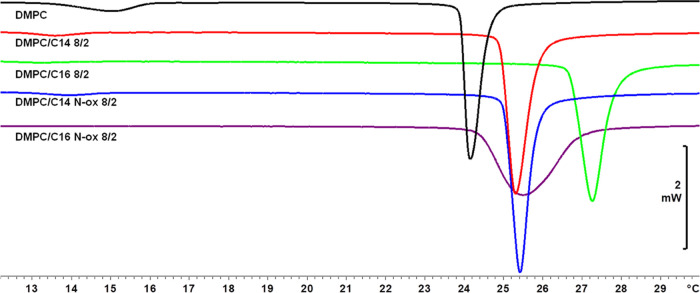
Thermograms of MLV containing DMPC or DMPC/l-prolinol derivative at 8/2 molar ratio. The scan rate is 1 °C/min.

The thermodynamic parameters and thermograms of the investigated liposomal formulations are reported in Table 3 and Figures 2 and 3. In the presence of synthetic surfactants, the main transition temperature (*T*_m_) was higher than the one observed for pure DMPC liposomes. In detail, the increase was higher for liposomes containing C**16** than for the ones with C**16**Nox in a concentration-dependent manner. No significant differences were observed among formulations containing C**14** and C**14**Nox. In the latter samples, the pretransition temperature (*T*_p_) always decreased and the peak appeared broader concerning the pure DMPC liposomes, whereas for C**16**- and C**16**Nox-containing liposomes the pretransition vanished.

**Table 3:** Thermodynamic Parameters Relative to the MLV Obtained by DSC Measurements^a^

Formulation	*T*_m_ (°C)	Δ*H*_m_ (kJ/mol)	CU	*T*_p_ (°C)
DMPC	24.1	18.6	75	15.3
DMPC/C**14** 8/2	25.3	24.7	45	13.6
DMPC/C**14**Nox 8/2	25.4	24.6	54	14.8
DMPC/C**16** 8/2	27.2	25.1	39	13.1
DMPC/C**16**Nox 8/2	25.5	22.7	17	
DMPC/C**14** 9/1	25.3	24.5	53	14.0
DMPC/C**14**Nox 9/1	25.1	22.7	48	13.5
DMPC/C**16** 9/1	26.1	23.4	36	12.4
DMPC/C**16**Nox 9/1	24.6	22.4	27	

a Uncertainty on the temperature is ±0.1 °C and that on Δ*H*_m_ is ± 0.5 kJ/mol.

The Δ*H* associated with the main transition was higher than the value observed with DMPC in all cases. The lowest values were observed in the presence of an *N*-oxide moiety at both molar ratios, except for DMPC/C**14**Nox 8/2.

The cooperative unit (CU) can be assessed to evaluate the cooperativity of the main transition. In general, the main transition of the liposome bilayer is highly cooperative: lipids move in unison with the surrounding molecules and start reorganizing well before *T*_m_. In other words, lipids cooperate and gain motional freedom: when a lipid undergoes the transition from gel to liquid-crystalline state, the nearby lipids gain motional energy and undergo the same transition easily. The efficiency of this effect increases at a temperature close to the *T*_m_ because the distance range of this cooperation enlarges. The larger the CU, the narrower the peak associated with the main transition (i.e., the temperature range in which the phase transition occurs). To summarize, this parameter represents an estimation of the number of lipids undergoing the phase transition simultaneously (i.e., at the same temperature) and can be obtained by the following equation
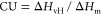
1where Δ*H*_vH_ indicates the van’t Hoff enthalpy change, an estimate of the enthalpy associated with the transition, which, based on the assumption of a simple two-state first-order transition model, can be considered as the amount of heat required for each cooperative unit to undergo the phase transition and is given by
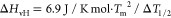
2The CU (Table 3) in mixed liposomes was lower than that in DMPC liposomes. In particular, the lowest values were observed with formulations containing C**16**Nox. In general, CU decreased with the increase of the chain length of the synthetic component.

### 4.3 *In Vitro* Susceptibility Test

The antimicrobial activity of liposomal formulations was evaluated by determining the MIC values. As shown in Table 4, all liposome formulations containing *N*-oxide surfactants did not exert activity against*E. coli* ATCC 25922 and *C. albicans*ATCC 64124, even at the highest concentration tested (MIC > 2.5 mM). According to the liposomal formulations, the Gram-positive strains revealed some differences: DMPC/C**16**Nox 9/1 formulation had no activity at the highest concentration, while DMPC/C**14**Nox 9/1 had a slightly better activity. DMPC/C**14**Nox 8/2 and DMPC/C**16**Nox 8/2 exhibited a good antimicrobial activity ranging from 0.078 to 0.625 mM (Table 4). On the other hand, cationic or pure DMPC liposomes did not exert any biological activity at the tested concentrations.

**Table 4:** Median MIC Values (mM) from Three Independent Experiments

	Formulation			
Microorganisms	DMPC/C**14**Nox 9/1	DMPC/C**16**Nox 9/1	DMPC/C**14**Nox 8/2	DMPC/C**16**Nox 8/2
*S. aureus* ATCC 43300	1.25	>2.50	0.625	0.156
*S. aureus* 29A	1.25	>2.50	0.313	0.313
*S. haemolyticus* 12H	1.25	>2.50	0.078	0.156
*S. epidermidis* 20E	1.25	>2.50	0.156	0.313
*E. coli* ATCC 25922	>2.50	>2.50	>2.50	>2.50
*C. albicans* ATCC 64124	>2.50	>2.50	>2.50	>2.50

Antibiotics belonging to four distinct classes, ERY (macrolide), CLI (lincosamide), GEN (aminoglycoside), and OXA (β-lactam), were also tested for their antimicrobial activity on *S. aureus* ATCC 43300. The MIC values of all antimicrobials were above their clinical resistance breakpoint.^34^ Antimicrobial susceptibility assay was also performed on *S. haemolyticus* 12H, *S. epidermidis* 20E, and *S. aureus* 29A to evaluate the antimicrobial activity of OXA. All three species were OXA resistant, with MIC values ranging from 1 to 256 μg/mL.

### 4.4 Checkerboard Microdilution Assay

The interactions between each antimicrobial and DMPC/C**14**Nox 8/2 and DMPC/C**16**Nox 8/2 were assessed by the two-dimensional checkerboard microdilution assay on the reference methicillin-resistant *S. aureus* ATCC 43300. The obtained data were analyzed with two nonparametric interpretative models, Loewe additivity-based model (Fractional Inhibitory Concentration Index, FICI) and Bliss independence-based model (Δ*E* model) (Tables 5, SI1, and SI2).^39^

**Table 5:** *In Vitro* Interactions between the Antibiotics and DMPC/C**14**Nox 8/2 and DMPC/C**16**Nox 8/2 against *S. aureus* ATCC 43300, Determined by the FICI^a^ Model and Δ*E*^b^ Model

		FICI		Δ*E* model		
Liposomes	Antibiotics	FICI_min_	INT	∑_SYN_ (*n*)	∑_ANT_ (*n*)	INT
DMPC/C**14**Nox 8/2	CLI	1	IND	949.3 (62)	–157.9 (15)	SYN
	ERY	1	IND	829.7 (69)	–85.4 (8)	SYN
	GEN	1	IND	1427.4 (62)	–145 (15)	SYN
	OXA	0.1563	SYN	730.4 (59)	–37.4 (18)	SYN
DMPC/C**16**Nox 8/2	CLI	1	IND	602.9 (50)	–60.5 (27)	SYN
	ERY	0.75	IND	937.7 (54)	–36.8 (23)	SYN
	GEN	1	IND	423.6 (35)	–334.6 (42)	SYN
	OXA	0.3125	SYN	823.3 (40)	–410.1 (37)	SYN

a INT, interpretation; SYN, synergy; ANT, antagonism; IND, indifference. SYN, FICI ≤ 0.5; ANT, FICI > 4; IND, FICI 0.5–4.

b INT, interpretation; SYN, synergy; ANT, antagonism; IND, indifference.

The interactions between DMPC/C**14**Nox 8/2 or DMPC/C**16**Nox 8/2 and CLY or ERY or GEN can be interpreted as simple additivity (0.5 < FICI < 4, indifference) with the Loewe model and synergism when analyzed with the Bliss model. On the other hand, OXA showed a clear synergy with the same liposomal formulations (DMPC/C**14**Nox 8/2 and DMPC/C**16**Nox 8/2) according to both interpretative models (Tables 5, SI1, and SI2).

The interactions between OXA and DMPC/C**14**Nox 8/2 or DMPC/C**16**Nox 8/2 were assessed by checkerboard microdilution assay on *S. haemolyticus* 12H, *S. epidermidis* 20E, and *S. aureus* 29A (Tables 6, SI3, and SI4). Synergism can be observed in all of these clinical strains between OXA and the formulations tested. The analytical models agree with each other, except for *S. epidermidis* 20E in the presence of DMPC/C**14**Nox 8/2.

**Table 6:** *In Vitro* Interactions between DMPC/C**14**Nox 8/2 and DMPC/C**16**Nox 8/2 in Combination with OXA against Two Clinical Species of CoNS and a Clinical *S. aureus*, Determined by the FICI^a^ Model and Δ*E*^b^ Model

		FICI		Δ*E* model		
Liposomes	Microorganism	FICI_min_	INT	∑_SYN_ (*n*)	∑_ANT_ (*n*)	INT
DMPC/C**14**Nox 8/2	*S. haemolyticus* 12H	0.5	SYN	467.8 (52)	–71.6 (24)	SYN
	*S. epidermidis* 20E	0.625	IND	522.8 (41)	–119.1 (34)	SYN
	*S. aureus* 29A	0.375	SYN	674.2 (40)	–140.3 (37)	SYN
DMPC/C**16**Nox 8/2	*S. haemolyticus* 12H	0.375	SYN	240.8 (29)	–160.0 (48)	SYN
	*S. epidermidis* 20E	0.5	SYN	486.7 (44)	–88.5 (32)	SYN
	*S. aureus* 29A	0.375	SYN	498.5 (31)	–89.8 (46)	SYN

a INT, interpretation; SYN, synergy; ANT, antagonism; IND, indifference. SYN, FICI ≤ 0.5; ANT, FICI > 4; IND, FICI 0.5–4.

b INT, interpretation; SYN, synergy; ANT, antagonism; IND, indifference.

## 5 Discussion

This paper aims to investigate the potential antimicrobial activity of liposomal formulations alone and in combination with commercially available antibiotics and correlate it with the molecular structure of the liposome components.

The liposomes under examination differ in the molar percentage composition, the carbon chain length, and/or the headgroup charge of their synthetic components (Table 1). As expected, all of the investigated formulations showed similar dimensions, and no significant differences are shown by the DLS diameters, as confirmed by the statistical analysis based on one-way ANOVA. The presence of a minor larger population is not surprising since it generally occurs upon sonication.^40^*Z*-potentials showed some interesting differences that are statistically significant (p < 0.05). Predictably, cationic liposomes featured systematic positive values, and for the same cationic surfactant, significantly larger values were observed by increasing the surfactant fraction. On the other hand, for liposomes containing *N*-oxide surfactants, the chain length influences *Z*-potential values. The relatively high value observed with DMPC/C**14**Nox formulations (despite the zwitterionic nature of the synthetic component) can be explained considering that the pyrrolidinium ring could be folded to expose the polar residues (*N*-oxide and OH groups) to the bulk, as observed in other systems containing analogue surfactants.^41^ The folding could be stabilized by the strong H-bond between the polar groups that typically occurs in *N*-oxide derivatives of l-proline.^42^ A systematic decrease of *Z*-potential values was observed by increasing the alkyl chain length (from C**14**Nox to C**16**Nox). This effect could be ascribed to a chain length mismatch, which is expected to bring variations in bilayer thickness and surface area in liposomes containing *N*-oxide surfactants.^43^ In our case, the mismatch between the DMPC chain length (14C) and that of C**16**Nox could cause a different exposure and/or counterion association of the polar headgroup, affecting the overall *Z*-potential of the aggregates. As a consequence, the *Z*-potentials decrease down to almost the value of the pure DMPC liposomes for the C**16**Nox containing ones and were comparable, independent of the surfactant fraction, within the ESD values.

Calorimetric data allow us to gain information about lipid organization. The inclusion of a synthetic surfactant in the DMPC bilayer causes better interactions in the polar region, as indicated by the increase of *T*_m_ and Δ*H* values (Table 3). Similar results were obtained in mixed liposomes composed of DMPC and l-prolinol derivatives analogous to those studied in this investigation (pH-sensitive and twin surfactants).^44^ Transformation of liquid-crystalline lipid bilayers into the gel state implies the formation of van der Waals contacts in the gel phase. The stronger these contacts, the higher the *T*_m_ and Δ*H* values. The observed increase of *T*_m_ denotes that the interactions between the nonpolar acyl chains are stronger than those in the bilayer of pure DMPC. Liposomes containing C**16**Nox show the largest peak associated with the main transition and the minor variation in Δ*H* values. This observation indicates that lipid packing is lower than the other investigated formulations (the phenomenon is also reflected in CU values).

Consequently, these pieces of evidence confirm the peculiarity of lipid interactions in the bilayer of DMPC/C**16**Nox liposomes and could partially explain their low *Z*-potential. In general, the increase of molar percentages of the synthetic components reduces CU, indicating that these molecules affect the organization of the bilayer. For instance, the disappearance of the pretransition in liposomes containing the longest surfactants indicates that a chain length mismatch disturbs the arrangement of the polar headgroups. Chain length mismatch can also affect *trans-gauche* chain isomerization and bilayer fluctuations in the case of liposomes formulated with *N*-oxide derivatives.^43^ Moreover, the effect of the chain length mismatch is more relevant if the bilayer contains a large excess of one of the components with respect to the other,^45^ as in the case of the investigated formulations. These effects could explain the different thermal behaviors observed between liposomes containing C14 and C16 synthetic lipids (thus containing surfactants that differ only for two methylenes in the alkyl chain). In similar previously investigated systems, we also observed differences based on different chain lengths: in the case of liposomes containing 10 molar percentage of CS or Nox surfactants bearing 12 methylene units in the alkyl chains, the stability of the formulations and the *Z*-potential, especially in the case of the C**12**Nox, are lower with respect to the C14 analogues.^46^

All of the liposomal formulations were tested on *S. aureus* MRSA ATCC 43300, *E. coli* ATCC 25922, and *C. albicans* ATCC 64124 to determine their respective MIC values. The extent of the antibacterial effect of the formulations depends on their composition. Surprisingly, cationic formulations did not exert any biological activity despite their relatively high Z-potential, often considered the only relevant parameter in cell interaction. Among the tested samples, DMPC/C**14**Nox 8/2 and DMPC/C**16**Nox 8/2 showed the greatest ability to inhibit the microbial growth against *S. aureus* ATCC 43300, with MIC values of 0.625 mM and 0.157 mM, respectively (Table 4). This result indicates that the *N*-oxide moiety plays a pivotal role in interacting with the biological environment, independently of the *Z*-potential. DMPC/C**14**Nox liposomes feature *Z*-potentials similar to the corresponding cationic formulations, whereas DMPC/C**16**Nox liposomes feature *Z*-potentials sensibly lower than those of DMPC/C**16** liposomes. It was also observed that the ability to inhibit microbial growth varies with the number of methylenes present in the l-prolinol derivative skeleton, in agreement with literature reports.^25,31,47,48^ In general, the higher the lipophilicity of the surfactant, the stronger the interactions with cell structures.^31,49^ These considerations can be simplistic. For instance, we observed that the influence of the formulations strongly depends on the nature of the microorganism. The variations in susceptibility can be explained considering the differences in *Z*-potential values and lipid organization that influence liposomes’ interaction with the cell walls of each strain.

As expected, the best results were obtained with the Gram-positive microorganisms, plausibly due to the absence of the outer membrane that favors the interaction between the liposomal formulations and the bacterial cell wall.^49^ Anyway, it is not astonishing that no inhibition towards the Gram-negative bacteria was observed. The low activity towards the eukaryote *C. albicans* certainly depends on the structural differences of the external fungal components. At the highest concentration (MIC > 2.5 mM), cytotoxic effects were not observed, hypothesizing that the formulations were not toxic.^50−52^

The antimicrobial activity shown towards *S. aureus* ATCC 43300 is particularly interesting because of the MDR phenotype that confers to this strain resistance to various classes of drugs: ERY (macrolide), CLI (lincosamide), GEN (aminoglycoside), and OXA (β-lactam). Therefore, it was decided to test liposomal formulations in combination with the antibiotics mentioned above to assess whether they can exert synergistic effects. It is important to highlight that the choice of the analysis model influences the interpretation of the drug’s interaction with the tested liposomes. There are many published methods for estimating drug–drug interactions.^53^ Among them, the FICI model based on the Loewe additivity theory and the Δ*E* model based on the Bliss independence theory were applied in this study. In general, we observed a reduction of the effective concentration of both liposomes and antibiotics in all of the cases.

For the FICI model, on observing the interaction between DMPC/C**14**Nox 8/2 and the antibiotics, only OXA was shown to exert synergistic effects. The strong interaction resulted in a 32-fold dose reduction for oxacillin and 16-fold reduction for the liposomal formulation (Tables 5 and SI2), so that the effective concentration of OXA turned out to be 0.5 μg/mL; therefore, it was possible to observe an induced phenotypic reversion of resistance toward the β-lactam antibiotic in *S. aureus*.

According to the 3D model, an agreement was found for the interaction with OXA, but the synergism was also observed with ERY. Specifically, it was possible to obtain a dose reduction of 512-fold for the antibiotic and 16-fold for DMPC/C**14**Nox 8/2 liposomes (Tables 5 and SI1). Furthermore, it allowed a decrease of the effective concentration of ERY up to 8 μg/mL, although it was impossible to fall below the clinical breakpoint value for this antibiotic. Frequently, the results from the two models were not completely consistent. For instance, the FICI considers only the effective combinations of the tested compounds, while the 3D model takes into account the sum of the differences (Δ*E*) between the experimental and the theoretical growth. Therefore, it can be considered as a global index of the combinative effect between the investigated compounds.

The combinations of DMPC/C**16**Nox 8/2 and the four antibiotics were consistent only for the interaction with OXA. In the FICI model, we could observe a 16-fold dose reduction for the β-lactam and a 4-fold decrease for the liposomes and in the 3D model, the synergism was found for all four antibiotics under examination. It is relevant to highlight that both the liposomal formulations drastically reduced the effective dose of OXA. It means that *S. aureus* becomes susceptible to a drug to which it shows resistance. These results are particularly interesting concerning OXA because the antibiotic is a β-lactam and defines the MRSA profile.

The antimicrobial activity of DMPC/C**14**Nox 8/2 and DMPC/C**16**Nox 8/2 was also tested against other staphylococci: specifically, two coagulase-negative species, *S. haemolyticus* 12H and *S. epidermidis* 20E, and a clinical *S. aureus* 29A. Their respective MIC values were determined as reported in Table 4. Since both formulations exhibited antimicrobial activities and the three organisms were resistant to OXA, the effects of their combinations were assessed (Tables 6, SI3, and SI4).

In the interaction between DMPC/C**14**Nox 8/2 or DMPC/C**16**Nox 8/2 and OXA (Tables 6, SI3, and SI4), a dose reduction of the antibiotic from 2 to 8 times was found, depending on the species. A significant result was observed towards *S. epidermidis* 20E, in which the effective concentration of the antibiotic turned out to be 0.125 μg/mL for DMPC/C**14**Nox 8/2 and 0.5 μg/mL for DMPC/C**16**Nox 8/2. The first combination dropped below the breakpoint value of OXA, while the second one was at the higher limit of its range. According to both methods, it is noteworthy that the relevant synergistic effect was observed only with OXA, which is a β-lactam antibiotic that hinders the synthesis of the peptidoglycan layer of bacterial cell walls by inhibiting the transpeptidase penicillin-binding proteins (PBPs). The lower β-lactam affinity mutant PBP2a, encoded by mecA, contributes to the methicillin resistance profile in *S. aureus* (MRSA). It is plausible that the investigated *N*-oxide formulations strongly interact with the bacterial membrane, influencing its organization and packing. The cell membrane’s disturbing action of the *N-*oxide formulations might influence the activity of PBP2a by altering the transmembrane domain of the enzyme. In the case of the other antibiotics, which exert their pharmacological action inside the cell, the combination with the same formulations is ineffective. If the liposomes were internalized, they could be disrupted in the cellular *milieu*, and thus they cannot synergistically support the drug action. Similar results were obtained on investigating the adjuvant potentialities of aggregates formulated with *N*-oxide surfactants without phospholipids combined with the same antibiotics against *S. aureus* MRSA.^25^

Overall, the results were certainly encouraging, although it was impossible to reach the breakpoint threshold for all drug combinations tested on all bacterial strains investigated. However, the ability of the liposomal formulations to interfere with cell growth is of great importance, even though they are not properly defined as antibiotics. The possibility to use them in adjuvant therapy, for example, in topical treatment,^54−56^ can be considered to improve the activity of antimicrobials (such as OXA) and to avoid the pharmacological resistance mechanisms. The main advantage of liposomes as an antimicrobial drug is that they do have not a specific target for interacting with cell structures. Liposomes act as chaotropic agents capable of determining the morpho-functional alterations of the membranes and consequent interferences with cell functions. The absence of a specific target is extremely important since it would avoid the development of resistance mechanisms toward these compounds. In addition, as the lipids employed in the liposome formulation substances have little or no toxicity,^25,57^ their use in adjuvant therapy would be safe.^53^ These results are undoubtedly positive, but still require further investigations to maximize the effect derived from each combination. In particular, it is possible to exploit the most promising formulations for their dual functions as drug carriers and antimicrobial agents.

## 6 Conclusions

The antibacterial activity of liposomal formulations alone and with different antibiotics was evaluated on several pathogens to investigate its correlation with liposomal composition. Our results point out that the presence of the *N*-oxide moiety is crucial, more than the charge of the formulations, to achieve the antibacterial effect. Moreover, lipid organization (strictly linked to the chain length of the synthetic components of the liposomes) plays a crucial role in determining the antimicrobial efficacy of liposomes. The effect is also strictly related to the nature of the microorganism and the specific target of the antibiotic. Based on these results, further investigations are ongoing on analogue formulations containing a higher molar percentage of *N*-oxide surfactants to improve the synergistic effect with antibiotic molecules and their efficacy on other microorganisms.

## Supporting Information Available

Additional data on *in vitro* interactions between DMPC/C**14**Nox 8/2 and DMPC/C**16**Nox 8/2 and antibiotics (PDF)
