# Supplementary material for: Structurally Related Liposomes Containing N-Oxide Surfactants: Physicochemical Properties and Evaluation of Antimicrobial Activity in Combination with Therapeutically Available Antibiotics
Source: Mol Pharm. 2022 Feb 16;19(3):788–97. doi: 10.1021/acs.molpharmaceut.1c00609 (PMC8905576; doi:10.1021/acs.molpharmaceut.1c00609)
Supplement: Supplementary file 1 — mp1c00609_si_001.pdf [file mp1c00609_si_001.pdf]

SUPPLEMENTARY INFORMATION

		ΔE Model			EFFECTIVE COMBINATION (ΔE_{MAX})		DOSE REDUCTION RATIO vs MIC	
**Liposomes**	**Antibiotic**	Σ_{SYN} (n)	Σ_{ANT} (n)	**INT**	**Antibiotic (µg/mL)**	**Liposomes (mM)**	**Antibiotic**	**Liposomes**
DMPC/C14Nox 8/2	***Clindamycin***	949.3 (62)	-157.9 (15)	SYN	1024	0.0394	16	16
	Erythromycin	829.7 (69)	-85.4 (8)	SYN	8	0.0394	512	16
	Gentamicin	1427.4 (62)	-145 (15)	SYN	64	0.0788	16	8
	Oxacillin	730.4 (59)	-37.4 (18)	SYN	4	0.0788	4	8
DMPC/C16Nox 8/2	***Clindamycin***	602.9 (50)	-60.5 (27)	SYN	64	0.0788	256	8
	Erythromycin	937.7 (54)	-36.8 (23)	SYN	2048	0.0788	2	8
	Gentamicin	423.6 (35)	-334.6 (42)	SYN	512	0.0788	2	8
	Oxacillin	823.3 (40)	-410.1 (37)	SYN	2	0.0394	8	16

[a]*INT*, interpretation; *SYN*, synergy; *ANT*, antagonism; *IND*, indifference

Table SI1. In vitro interactions between DMPC/C14Nox 8/2 and DMPC/C16Nox 8/2 and antibiotics against *S. aureus* ATCC 43300, determined by ΔE^{a} model

				EFFECTIVE COMBINATION		DOSE REDUCTION RATIO vs MIC	
**Antibiotic**	**Liposomes**	**$FICI_{min}$**	**INT**	**Antibiotic (µg/mL)**	**Liposomes (mM)**	**Antibiotic**	**Liposomes**
Oxacillin	DMPC/C14Nox 8/2	0.1563	SYN	0.5	0.0394	32	16
	DMPC/C16Nox	0.3125	SYN	1	0.0394	16	4

	8/2						

[a]INT, interpretation; SYN, synergy; ANT, antagonism; IND, indifference. SYN FICI ≤0.5; ANT, FICI> 4; IND, FICI 0.5-4

Table SI2. In vitro interactions between oxacillin and both DMPC/C14Nox 8/2 and DMPC/C16Nox 8/2 against *S. aureus* ATCC 43300, determined by FICI[a] model

				EFFECTIVE COMBINATION		DOSE REDUCTION RATIO vs MIC	
Liposomes	Microorganism	$FICI_{min}$	INT	Antibiotic (µg/mL)	Liposomes (mM)	Antibiotic	Liposomes
DMPC/C14Nox 8/2	*S.haemolyticus* 12 H	0.5	SYN	64	0.0195	2	4
	S.epidermidis 20 E	0.625	IND	0.125	0.0781	8	2
	S. aureus 29 A	0.375	SYN	32	0.0781	8	4
DMPC/C16Nox 8/2	*S.haemolyticus* 12 H	0.375	SYN	16	0.0394	2	4
	S.epidermidis 20 E	0.5	SYN	0.5	0.0781	8	4
	S. aureus 29 A	0.375	SYN	16	0.0781	8	4

[a]INT, interpretation; SYN, synergy; ANT, antagonism; IND, indifference. SYN FICI ≤0.5; ANT, FICI> 4; IND, FICI 0.5-4

Table SI3. In vitro interactions between oxacillin and both DMPC/C14Nox 8/2 and DMPC/C16Nox 8/2 against two clinical strain of CoNS and a clinical *S. aureus*, determined by FICI[a]

					EFFECTIVE COMBINATION (ΔE_{MAX})		DOSE REDUCTION RATIO vs MIC	
Liposomes	Microorganism	Σ_{SYN} (n)	Σ_{ANT} (n)	INT	Antibiotic (µg/mL)	Liposomes (mM)	Antibiotic	Liposomes
DMPC/C14 Nox 8/2	*S.haemolyticus* 12 H	467.8 (52)	-71.6 (24)	SYN	32	0.0390	4	2

	S.epidermidis 20 E	522.8 (41)	-119.1 (34)	SYN	0.5	0.0390	2	4
	S. aureus 29 A	674.2 (40)	-140.3 (37)	SYN	32	0.0781	8	4
DMPC/C16 Nox 8/2	*S.haemolyticus* 12 H	240.8 (29)	-160.0 (48)	SYN	4	0.0781	4	2
	S.epidermidis 20 E	486.7 (44)	-88.5 (32)	SYN	0.25	0.0781	2	4
	S. aureus 29 A	498.5 (31)	-89.8 (46)	SYN	32	0.0781	8	4

[a]INT, interpretation; SYN, synergy; ANT, antagonism; IND, indifference

Table SI4. In vitro interactions between oxacillin and both DMPC/C14Nox 8/2 and DMPC/C16Nox 8/2 against two clinical strain of CoNS and a clinical *S. aureus*, determined by ΔE[a] model
